# Niche partitioning and the role of intraspecific niche variation in structuring a guild of generalist anurans

**DOI:** 10.1098/rsos.170060

**Published:** 2017-03-15

**Authors:** Carl S. Cloyed, Perri K. Eason

**Affiliations:** 1 National Great Rivers Research and Education Center, East Alton, IL 62024, USA; 2 Department of Biology, Washington University in St Louis, St Louis, MO 63130, USA; 3 Department of Biology, University of Louisville, Louisville, KY 40292, USA

**Keywords:** coexistence, frogs, intra-population niche variation, individual specialization, niche partitioning, toads

## Abstract

Intra-population niche differences in generalist foragers have captured the interest of ecologists, because such individuality can have important ecological and evolutionary implications. Few researchers have investigated how these differences affect the relationships among ecologically similar, sympatric species. Using stable isotopes, stomach contents, morphology and habitat preference, we examined niche partitioning within a group of five anurans and determined whether variation within species could facilitate resource partitioning. Species partitioned their niches by trophic level and by foraging habitat. However, there was considerable intraspecific variation in trophic level, with larger individuals generally feeding at higher trophic levels. For species at intermediate trophic levels, smaller individuals overlapped in trophic level with individuals of smaller species and larger individuals overlapped with the smallest individuals from larger species. Species varied in carbon isotopes; species with enriched carbon isotope ratios foraged farther from ponds, whereas species with depleted carbon isotope values foraged closer to ponds. Our study shows that these species partition their niches by feeding at different trophic levels and foraging at different distances from ponds. The intraspecific variation in trophic level decreased the number of individuals from each species that overlapped in trophic level with individuals from other species, which can facilitate species coexistence.

## 1. Introduction

Ecologically similar, sympatric species may avoid competitive exclusion by partitioning their niches [1–4]. Niche partitioning has been a primary focus of many population and community ecology studies, as it resolves the paradox between early theoretical/laboratory studies demonstrating competitive exclusion and the fact that many ecosystems have ecologically similar species that do not drive one another extinct [5–7]. Several classic ecological studies investigated how similar species can coexist (Homage to Santa Rosalia and the Hutchinsonian niche) [6–8], how such species can divide seemingly homogeneous resources (MacArthur's warblers and the paradox of the plankton) [1,7] and how similar two species can be before one starts to exclude the other (limiting similarity) [9]. Thus, niche partitioning has been a central idea in ecology for over half a century, and it remains an active and important part of ecological research [4,10–12].

Species partition resources in a variety of ways, most often taking advantage of environmental heterogeneity. First, similar species often feed in different habitats and microhabitats [13–19]. For example, Arctic charr, *Salvelinus alpinus*, and brown trout, *Salmo trutta*, feed at different depths in Norwegian lakes [17], cottid fishes forage on different substrates in sub-tidal habitats along the Washington state coast [18], and several species of North American warblers feed at different heights and distances from the trunk within the same trees [1]. Species can also partition resources by foraging on different items [16,19–22], and combining isotopic data with traditional foraging and morphological data has enhanced our ability to understand sometimes subtle species differences in food resources. Stable isotope and stomach content analyses showed that two species of cod, *Gadus morhua* and *G. ogac*, feed on different prey items but at the same trophic levels [22]. Stable isotope and stomach content analyses also showed that tropical, freshwater fishes track changes in food abundance across season, and morphological analyses further demonstrated that they prefer food items that they can ingest most efficiently [12]. Some species divide resources temporally, either by having different daily activity periods or through seasonal differences in foraging [10,20–26]. For example, the common spiny mouse, *Acomys cahirinus*, has shifted to foraging nocturnally due to the diurnally foraging golden spiny mouse *Acomys russatus*, and two sympatric sparrows partition diets by foraging at different trophic levels, but only in seasons when resource availability is high [19]. The use of different foraging methods or tactics can also be a means of partitioning resources [3,24]. Tropical leaf-litter anurans have one of two primary foraging strategies, active pursuit and sit-and-wait, and anurans using each strategy differ in diet from those using the other [3]. Finally, species can partition resources in more than one way. For example, pumpkinseed sunfish, *Lepomis gibbosus*, and longear sunfish, *Lepomis megalotis*, prefer habitats with different degrees of vegetative cover but also prefer different prey [27]. Desert bat communities and East African carnivore communities partition hunting both spatially and temporally [28,29], and cormorants (*Phalacrocorax niger*, *P. fuscicollis* and *P. carbo*) partition foraging temporally or by habitat [30].

Although the above and many other studies demonstrate the importance of niche partitioning, few consider the role that intraspecific trait variation may play in partitioning resources among species [31,32]. Recent studies on generalist foragers have demonstrated that individuals of some of these species act as specialists that consume only a subset of the species' resource spectrum [32–39]. These intraspecific trait variations can take many forms [32–35], including variation between sexes [34,35], among individuals [26,36–39] and across body sizes or ontogeny [23,40–43]. These kinds of intraspecific variation can have important ecological and evolutionary consequences [41]. For example, intraspecific trait variations can increase population stability through the portfolio effect [44] or through reduced intraspecific competition [37,38,42]. Individual trait variation may also promote species coexistence by decreasing the number of individuals from each species that overlap in niche space, thereby blurring niche boundaries and allowing for more overlap among species [31,40,45,46]. Studies on ontogenic changes in niche have found that resource overlap among groups of species may change across different life stages [47–50] and that, as a result, only a certain subset of individuals may be competing with individuals from another species at any given time [47,50]. Considering individual trait variation may help clarify the results of some studies of niche partitioning in which species differences were difficult to detect [31].

We investigated resource partitioning and intraspecific trait variation in five anurans, *Anaxyrus americanus, A. fowleri, Lithobates catesbeianus, L. clamitans* and *L. sphenocephalus*. There are two reasons why these frogs and toads represent an excellent system in which to investigate resource partitioning and the role of intraspecific trait variation in species coexistence. First, these species have large dietary overlap [51–55]. Although competition has not been studied across all five species, some research has addressed competition between species pairs within the group. *Lithobates catesbeianus* is more likely to be found in ponds and to take a greater proportion of aquatic prey than *L. clamitans* [55]. Additionally, *L. catesbeianus* is a superior competitor to *L. clamitans* [56], but in the laboratory *L. clamitans* is competitively superior to *A. americanus* [57]. Second, these anurans are generalist foragers [51–55] and therefore offer an opportunity to investigate the role of intraspecific niche variation in facilitating niche partitioning. Studies on generalist frog species have found evidence of intraspecific niche variation [39,58], and several species of frogs, including *L. catesbeianus*, increase their trophic level with increasing snout–vent length (SVL) [41].

We used a multifaceted approach to assess niche partitioning and to investigate the possible role of intra-population niche variation in this partitioning. We predicted that each of these frog and toad species would differ in their niche from each of the other species along at least one axis, whether in prey preference, trophic level or habitat use. Furthermore, we predicted that individual-level niches would vary within species, decreasing the number of individuals among heterospecifics that might overlap in resource use, thereby decreasing the interaction strength and aiding resource partitioning. To measure resource use and diets, we analysed both stable isotope and stomach content data. Additionally, we measured morphological traits to test whether resource use and diet patterns correlated with morphology, and we measured how far frogs and toads were found from ponds to assess spatial partitioning. We used the morphological variables to determine whether individuals' body shape enabled individual-level differences in diet and whether those morphologically driven differences in diet were conducive to species coexistence. The combination of two measures of diet with data on morphology and habitat use enabled us to investigate the complex ways that niche partitioning occurs and how individual differences can affect that niche partitioning.

## 2. Material and methods

### 2.1. Study site

This study was performed at ten small, permanent ponds in Bernheim Arboretum and Research Forest, Clermont, Kentucky, USA (we obtained permission from Bernheim staff to collect individuals and carry out our project). Bernheim is located in the Knobs region of Kentucky (37°55′20.84^″^ N, 85°39′25.85^″^ W). Ponds ranged from 41 to 1329 m^2^ in size, were shallow (less than 1.5 m deep) and in general had gentle slopes at their margins. Eight ponds were in the forest interior and two ponds were at the transition between a forest and small grassland. In general, these ponds clustered into four different regions (electronic supplementary material, figure S1), and ponds within each cluster were close enough together (less than 2 km) that some dispersal occurred among the ponds (C. S. Cloyed 2014, personal observation)[59–61]. White oak–black oak, *Quercus alba–Q. velutina*, and white oak–chestnut oak, *Q. alba–Q. prinus*, were the most common forest types. The most abundant understorey plants were *Smilax* spp., *Toxicodendron radicans, Leersia oryzoides* and *Microstegium vimineum.* Predators of adult frogs that are found around these ponds include raccoons, *Procyon lotor*, and coyotes, *Canis latrans.*

### 2.2. Frog and toad capture and processing

We collected frogs and toads from April through September in 2011 and 2012 and from April to June in 2013 and 2014. *Anaxyrus americanus* individuals were captured from 2011 to 2014, and *A. fowleri* individuals were captured in 2013 and 2014. Both toad species were mostly captured in the spring and early summer, when they were more common at the field sites; they were almost never found near the ponds, as they only visit ponds to breed and most breeding occurred earlier in the year than when this study was performed. We captured *Lithobates sphenocephalus* individuals during 2011–2014 and primarily in spring and early summer, when they were common around the ponds and before they had migrated away from the ponds subsequent to the breeding season. *Lithobates sphenocephalus* was captured from pond banks as well as in the forests and grasslands surrounding the ponds. Both *L. catesbeianus* and *L. clamitans* were captured during 2011–2014 throughout the springs and summers, as they remained near the ponds during both seasons. In general, *L. catesbeianus* and *L. clamitans* were collected around the pond banks, where both calling and non-calling individuals were captured. We used hand nets which had approximately 1.5 m long handles to capture frogs and toads in and around ponds starting 30 min after sunset and continuing for an average of 55 min. The general shape of the ponds and their shallow depth combined with the long handles on our nets allowed us to sample up to 3 m into the pond. Two people worked together to collect frogs and toads, with one person working at a pond and the other in the woods or fields surrounding it. When frogs and toads were captured, their initial location was marked and they were placed in plastic containers. Each evening after frogs and toads were collected, we measured the distance from their initial locations to the pond edge and recorded that distance as a positive number if they were outside the pond and as a negative number if they were in the pond.

We measured morphological variables with a Swiss Precision Instruments dial caliper to the nearest millimetre. To test whether morphology was correlated with diet, we measured morphological variables relating to feeding: gape width and mandible length [62]. We also measured morphological variables related to jumping: SVL, the lengths of the right femur, tibia, and the combined length of the metatarsals and fourth phalanx [62]. We additionally created the variable leg length by summing the lengths of the femur, tibia and metatarsal/phalanx.

We obtained stomach contents with a gentle stomach flushing technique [63]. We caught any expelled stomach contents in a cup and stored them in 95% ethanol. Prey were identified later in the laboratory. Prey groups for stomach content analysis (SCA) included orthopterans, ants, coleopterans, miscellaneous non-flying prey and miscellaneous flying prey. Miscellaneous non-flying prey included spiders, flightless insects from the families Pentatomidae, Reduviidae and Membracidae, other non-flying hemipterans and larval lepidopterans. The miscellaneous flying prey group included flying hymenopterans, dipterans, flying hemipterans such as cicadellids and cercopids, adult lepidopterans and adult odonates. We divided stomach contents into these prey groups because of their frequency in the stomachs of our focal species.

Frogs and toads were individually marked with a unique combination of toe clips. Skin from these toe clips was used for SIA. Clipped toes were placed in a chilled cooler in the field, dried for 48 h at 60°C and then stored in a cool, dark drawer in the laboratory. After the toe clip, frogs were released where they were captured.

### 2.3. Stable isotope analysis

After samples were dried, we manually separated the bone of the toe from skin and ligaments with an Xacto^©^ knife. Each skin sample was weighed to between 0.3 and 0.7 mg in tin capsules on a Mettler Toledo AG245 micro-scale. Samples were analysed at the University of New Mexico's Center for Stable Isotopes. The samples were combusted in a Costech 4010 elemental analyser (Costech, Valenicia, CA, USA) coupled to a Thermo Scientific Delta V mass spectrometer (Thermo Scientific, Bremen, Germany).

Stable isotope values were expressed with standard delta notation (δ) in parts per thousand (‰), where δ*X* = (*R*_sample_/*R*_standard_ − 1) × 1000, where *X* is δ^13^C or δ^15^N and *R*_sample_ and *R*_standard_ are the molar ratios of C^13^/C^12^ and N^15^/N^14^ of the sample and the standard reference material, respectively. The reference material was Vienna-Pee Dee Belemnite for carbon and atmospheric N_2_ for nitrogen.

### 2.4. Prey collection

To determine local abundances of prey species, we collected arthropods around a pond within 10 days of sampling frogs or toads at that pond. To collect ground-dwelling arthropods, we placed 7.62 cm diameter pit-fall traps on a 2 m wide transect that began at the pond's edge and continued 130 m into the surrounding habitat. Two pit-fall traps were placed 0–2 m, 13–15 m, 28–30 m, 113–115 m and 128–130 m from each pond; at each of these five sites on a transect, the two traps were placed on opposite sides of the transect line and 1–2 m apart. All traps were left open for 48 h, after which the contents were collected. Within a few minutes after collecting contents from pit-fall traps, we took sweep-net samples to collect flying arthropods and arthropods in foliage close to the ground. Sweep netting took place at 0, 15, 30, 115 and 130 m and used 20 of C.S.C.'s sweep-steps at each location, which covered an area approximately 1.5 × 20 m. Collected arthropods were taken to the laboratory for identification. We summed prey from all sweep-net and pit-fall traps for each pond, calculated the proportion of prey constituted by each prey category and used these proportions as estimates of the relative frequency of prey types in the environment.

### 2.5. Statistical analyses

We used principal components analysis (PCA) to organize morphological variation and make adjustments to morphological data to account for differences in body size [64]. To make these size adjustments, we first performed a preliminary PCA with all morphological variables including SVL, which resulted in a first PCA axis that loaded along the SVL. We then regressed the remaining non-size morphological variables against the values from the first axis of the preliminary PCA and used the residuals from this regression as the new variables in the final PCA [64]. We used ANOVA, or the Kruskal–Wallis test when the data failed to meet the assumptions of normality and equal variances, to determine differences among species in the first two axes produced in the PCA, SVL, and distance to pond. To test for pairwise differences between species in these traits and in distance to pond, we used Tukey's honest significant differences test when an ANOVA was used and pairwise Wilcoxon tests with Bonferroni-adjusted *p*-values when a Kruskal–Wallis test was used.

We used two general linear mixed-effects models (GLMMs) to test for differences in carbon and nitrogen isotopes. For these models, carbon or nitrogen isotopic values were the dependent variable, and species, year, sex, the first two axes from the size-adjusted PCA, season (spring or summer) and SVL were the independent variables. We included SVL because frogs are known to change their diets as they grow [25,41]. We included season × species and SVL × species interactions to test whether species’ isotopic values differed between seasons and whether isotopic values changed with SVL differently among species. To determine whether isotope baseline values varied geographically, we included region in the initial model. Ponds were grouped by proximity into four regions, North, North Central, South Central and South. There were three ponds in the North region, two ponds in the North Central region, three ponds in the South Central region and two ponds in the South region (electronic supplementary material, figure S1). Based on typical species ranging distances, frogs could have moved to different ponds within regions but were unlikely to travel among regions [59–61]. If region was significant, then we nested further analyses by region. We used a combination of AIC_c_ and *p*-values to determine the model that best fit the data. As is standard procedure, models with a ΔAIC_c_ of greater than 2 were dropped and parameters with *p-*values greater than 0.05 were dropped. To determine whether individuals from each species could be distinguished from individuals of other species based on isotope values, we used hierarchical clustering analysis for each isotope type to group individuals from all species and then ran linear models in which group number was the response variable and species was the explanatory variable. To corroborate the above GLMMs performed on the raw isotope data, we ran additional linear models in which group number was the explanatory variable and the response variables were the final response variables from the analysis on the raw isotope data.

We calculated Chesson's alpha [65] on prey groups for SCA. Chesson's alpha compares prey frequency in stomachs to prey frequency in the environment and thus is a measure of selectivity [65]. Chesson's alpha ranges from 0 to 1, where 0 represents complete avoidance of a prey type and 1 represents complete preference for a prey type. Given that we had five prey groups, a Chesson's alpha of 0.2 for a particular prey group implies that its frequency in stomach contents matches its frequency in the environment. We used a perMANOVA in the R package Vegan [66] to determine differences among species. This perMANOVA was done with 50 000 permutations using the Bray distance. We followed this perMANOVA with permutation tests between species to determine which species differed significantly. These tests were done with 50 000 permutations using Markov chain Monte Carlo methods. All statistical analyses were done in R [67].

## 3. Results

### 3.1. Morphology and distance to pond

We performed PCA on 53 *A. americanus*, 38 *A. fowleri*, 61 *L. catesbeianus*, 75 *L. clamitans* and 39 *L. sphenocephalus*. In the PCA, both the first and second axes explained 17% of the morphological variation (electronic supplementary material, table S1). The first PCA axis loaded most strongly with leg length (0.51), and the second axis loaded most strongly with metatarsal length (0.86). There were significant overall differences among species in the first PCA axis, associated with size-adjusted leg length (*F* = 155.1, d.f. = 4, 261, *p* < 0.001). We used Tukey's honest significant differences test to determine pairwise differences between species. For the first PCA axis, these differences were not significant between the two toads, *A. americanus* and *A. fowleri*, or between *A. fowleri* and *L. catesbeianus,* but were significant for all other pairwise species combinations (*p* < 0.001 for all comparisons). Species also differed in the second PCA axis, associated with size-adjusted metatarsal length (*F* = 5.035, d.f. = 4, 261, *p* < 0.001); *L. catesbeianus* had smaller size-adjusted metatarsal length than *L. clamitans* (*p* = 0.002) and both toad species (*p* = 0.008 between *A. americanus* and *L. catesbeianus* and *p* = 0.015 between *A. fowleri* and *L. catesbeianus*). There were no significant differences between any other species pair.

**Figure 1.**
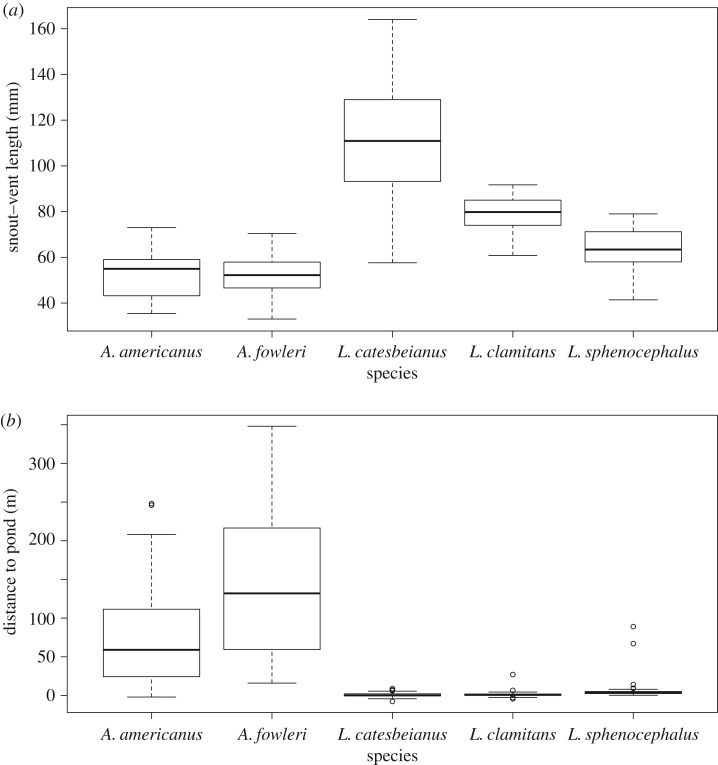
(*a*) SVL (mm) by species. *Anaxyrus americanus* and *A. fowleri* were the smallest species, followed by *Lithobates sphenocephalus* and *L. clamitans*. *Lithobates catesbeianus* was the largest species but also had the largest variance, with individuals falling within the body size range of all other species. (*b*) Distance to pond (m) by species. Both toad species are infrequently found near ponds and *A. fowleri* generally are found farther from ponds than *A. americanus*. *Lithobates clamitans* and *L. catesbeianus* have about an equal distance to pond, with both species frequently found within ponds (negative numbers). *Lithobates sphenocephalus* is found farther from ponds than the other two frog species but nearer ponds than both toad species.

Species differed in SVL (ANOVA: figure 1*a*; *F* = 119.3, d.f. = 4,237, *p* < 0.001). Tukey's honest significant differences test showed that *L. catesbeianus* was longer than the other four species (*p* < 0.001 for all comparisons). In addition, *L. clamitans* was longer than *L. sphenocephalus* (*p* = 0.003), *A. anaxyrus* (*p* < 0.001) and *A. fowleri* (*p* < 0.001), and *L. sphenocephalus* was longer than the two toads (*A. americanus: p* = 0.053; and *A. fowleri: p* = 0.013).

Distance to pond edge varied significantly among species (figure 1*b*; Kruskal–Wallis test: *χ*^2^ = 212.18, d.f. = 4, *p* < 0.001). Pairwise Wilcoxon tests showed that both *A. fowleri* and *A. americanus* toads were found farther from ponds than were any of the three frog species (*p* < 0.001 for all comparisons). *Lithobates sphenocephalus* occupied sites at greater distance from ponds than either *L. catesbeianus* or *L. clamitans* (*p* < 0.001 for both comparisons).

### 3.2. Stable isotope analysis

**Figure 2.**
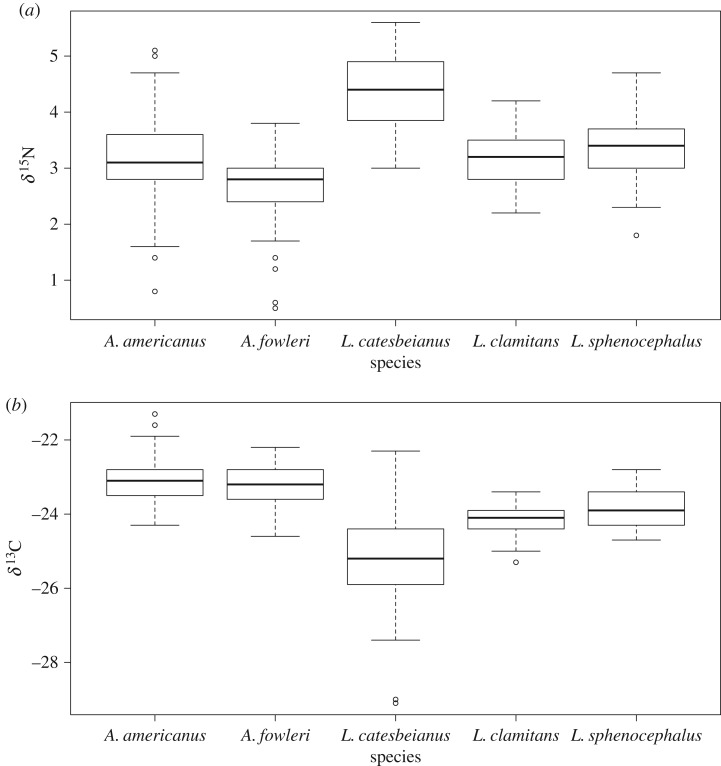
(*a*) Box and whisker plot of δ^15^N values by species. *Anaxyrus americanus*, *Lithobates clamitans* and *L. sphenocephalus* have similar δ^15^N values, while *L. catesbeianus* has enriched δ^15^N values and *A. fowleri* has depleted values. (*b*) Box and whisker plot of δ^13^C values by species. *Anaxyrus americanus* and *A. fowleri* have similar δ^13^C values; *L. clamitans* and *L. sphenocephalus* have similar δ^13^C values; *L. catesbeianus* has depleted δ^13^C values but also has the greatest variance and overlaps with most of the ranges of δ^13^C values for all other species.

We collected samples for stable isotope analysis on 53 *A. americanus* (year (*N*): 2011 (17), 2012 (10), 2013 (14) and 2014 (12); season (*N*): spring (48), summer (5)), 38 *A. fowleri* (2013 (20), 2014 (18); spring (33), summer (5)), 61 *L. catesbeianus* (2011 (17) 2012 (33), 2013 (2), 2014 (9); spring (21), summer (40)), 75 *L. clamitans* (2011 (34), 2012 (41); spring (33), summer (42)) and 39 *L. sphenocephalus* (2011 (14), 2012 (19), 2013 (1), 2014 (5); spring (14), summer (25); electronic supplementary material, table S2). Isotopic values varied among regions for both carbon (*F* = 3.271, d.f. = 3, 261, *p* = 0.012) and nitrogen (*F* = 13.242, d.f. = 3, 261, *p* < 0.001), and we therefore nested further analyses by region. Electronic supplementary material, table S3 shows the complete list of models generated for both carbon and nitrogen isotopes. For nitrogen, two models had low AIC_c_ values, one with SVL (*F* = 33.57, d.f. = 1, 256, *p* < 0.001) and species (*F* = 52.63, d.f. = 4, 256, *p* < 0.001) and the second with SVL (*F* = 33.90, d.f. = 1, 255, *p* < 0.001), species (*F* = 53.15, d.f. = 4, 255, *p* < 0.001) and season (*F* = 3.58, d.f. = 1, 255, *p* = 0.060). However, since season had only a marginally significant *p*-value, it was not included in the final model, and therefore the best fitting model for nitrogen included species and SVL. Nitrogen isotope values differed among species, with *L. catesbeianus* having the most enriched values. The other two true frogs, *L. clamitans* and *L. sphenocephalus*, had similar nitrogen isotope values that were intermediate between values for *L. catesbeianus* and for the two toads, which had similar values (figure 2*a*). The model of best fit for carbon included only species (*F* = 79.20, d.f. = 4, 256, *p* < 0.001) and SVL (*F* = 44.12, d.f. = 1, 256, *p* < 0.001). The two toads, *A. americanus* and *A. fowleri*, had the most enriched carbon isotope values, followed by *L. sphenocephalus* and *L. clamitans* (figure 2*b*). *Lithobates catesbeianus* had the most depleted carbon isotope values (figure 2*b*).

**Figure 3.**
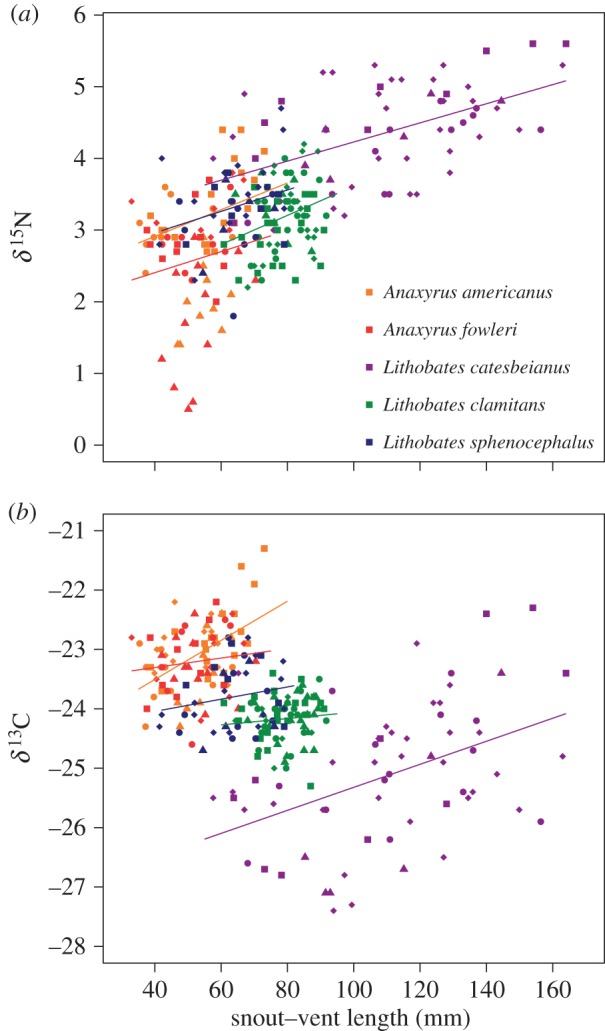
(*a*) δ^15^N values and (*b*) δ^13^C values and versus SVL for each species. Each species is represented by the colour in legend in panel (*a*). Lines for each species are represented by the same colour symbol and are the general linear models. Increasing δ^15^N values with SVL within species demonstrates how intraspecific variation can aid resource partitioning, as overlap in trophic niche is size-dependent. Smaller individuals within a species overlap in trophic niche with a different set of other species than larger individuals. This is particularly pronounced in *L. clamitans* and *L. catesbeianus*. Each region is represented by a differently shaped symbol: North (squares), North Central (circles), South (triangles), South Central (diamonds). Lines represent the slope of the regression without being nested for each site.

To examine the relationship between isotope types and SVL for each species, we used GLMM in which region was a random factor. When differences in isotope values among regions had been accounted for, individuals of *A. americanus*, *L. clamitans* and *L. catesbeianus* with longer SVL had more enriched δ^15^N values (table 1 and figure 3*a*). However, SVL did not have a significant effect on δ^15^N values in *A. fowleri* or *L. sphenocephalus* (table 1 and figure 3*a*). Individuals of *A. americanus* and *L. catesbeianus* with longer SVL also had more enriched δ^13^C values (table 1 and figure 3*b*), but SVL did not significantly affect δ^13^C values in *A. fowleri*, *L. sphenocephalus* or *L. clamitans* (table 1 and figure 3*b*).

**Table 1.** Statistical values for GLMM testing whether isotopic values vary by SVL nested by region.

	*t*	d.f.	*p*-value
δ^15^N			
*A. americanus*	3.123	47	<0.006
*A. fowleri*	1.528	33	0.136
*L. catesbeianus*	5.000	56	<0.001
*L. clamitans*	2.542	70	0.013
*L. sphenocephalus*	1.813	34	0.079
δ^13^C			
*A. americanus*	3.683	47	<0.001
*A. fowleri*	0.741	33	0.464
*L. catesbeianus*	3.720	56	<0.001
*L. clamitans*	0.847	70	0.400
*L. sphenocephalus*	1.157	34	0.255

**Figure 4.**
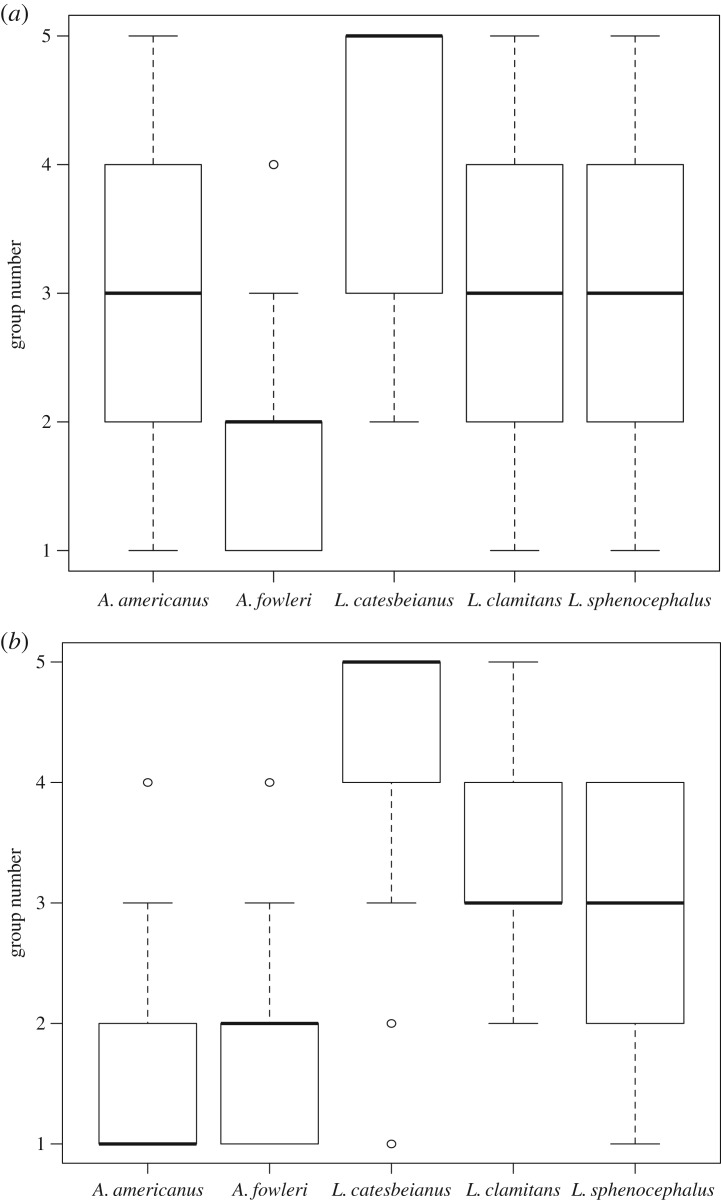
Group membership for (*a*) δ^15^N and (*b*) δ^13^C by species as determined by hierarchical clustering. Group number indicates which group an individual frog was placed in according to the clustering analysis. For both isotope types, *L. catesbeianus* is most frequently found in higher-numbered groups. For δ^15^N, *A. americanus, L. clamitans* and *L. sphenocephalus* most frequently cluster in intermediate-numbered groups but are present in all groups. *Anaxyrus fowleri* cluster most frequently in lower-numbered groups. For δ^13^C, both *A. americanus* and *A. fowleri* cluster in lower-numbered groups. *Lithobates sphenocephalus* cluster in intermediate-numbered groups, and both *L. catesbeianus* and *L. clamitans* cluster in higher-numbered groups. The clustering pattern for δ^13^C generally follows the pattern of distance from pond at which each species was found, as shown in the electronic supplementary material, figure S2*b*.

Individuals that were grouped by δ^15^N values in a cluster analysis differed according to species (figure 4*a*; *F* = 36.76, d.f. = 4, 261, *p* < 0.001), and in general larger individuals were placed in higher-numbered groups (electronic supplementary material, figure S2*a*). Individuals from *A. americanus*, *L. clamitans* and *L. sphenocephalus* did not differ in the groups to which they belonged (figure 4*a*). Individuals from *L. catesbeianus* were grouped higher than all other species (*p* < 0.001 for all comparisons) but did overlap with some individuals from *A. americanus, L. clamitans* and *L. sphenocephalus* (figure 4*a*). Individuals from *A. fowleri* were grouped lower than the other species (figure 4*a*; *p* < 0.001 for all comparisons), but some individuals overlapped with *A. americanus, L. clamitans* and *L. sphenocephalus* (figure 4*a*). Individuals from higher groups had higher SVL (*F* = 105.2, d.f. = 1, 264, *p* < 0.001, *R*^2^ = 0.282).

Individuals grouped by δ^13^C values in a cluster analysis also differed according to species (figure 4*b*; *F* = 69.02, d.f. = 4, 261, *p* < 0.001). *Anaxyrus americanus* and *A. fowleri* did not differ in the group to which they most often belonged (*p* = 0.994), nor did *L. clamitans* and *L. sphenocephalus* (*p* = 0.086). All other species combinations differed (*p* < 0.001 for all comparisons). Individuals from different groups were found at different distances from the pond, and because the effects of species on group number were strong, a graph of these results nearly mirrored figure 1*b* (electronic supplementary material, figure S2*b*).

### 3.3. Stomach content analysis

**Figure 5.**
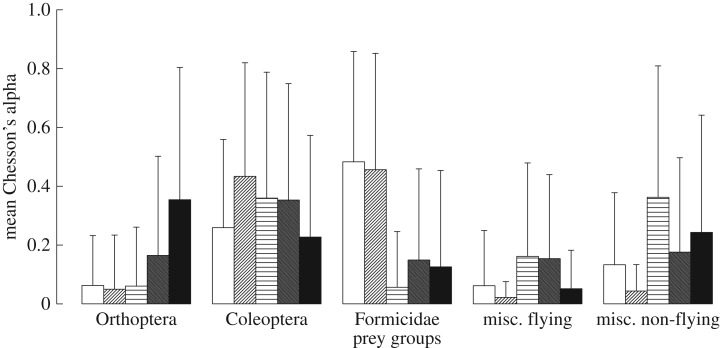
Mean Chesson's alpha (±s.d.) depicting prey preference for each prey group across all five species of frogs and toads. In our case, a Chesson's alpha of 0.2 indicates that frogs and toads are taking prey in proportion to how common they are in the environment. Values above 0.2 indicate preference for that prey group and values below 0.2 indicate avoidance of that prey group. All species prey upon beetles about equally and all species prey upon miscellaneous non-flying prey more than miscellaneous flying prey. The two toad species, *A. americanus* and *A. fowleri,* prey upon ants the most. *Lithobates sphenocephalus* prey upon orthopterans more than do the other species. Grouping left to right: *A. americanus, A. fowleri, L. catesbeianus, L. clamitans* and *L. sphenocephalus*.

We obtained stomach contents from 36 *A. americanus*, 26 *A. fowleri*, 39 *L. catesbeianus*, 100 *L. clamitans* and 30 *L. sphenocephalus*. The Chesson's alpha values differed significantly among species (electronic supplementary material, table S4, and figure 5; *F* = 6.481, d.f. = 4, 207, *p* < 0.001). In general, both toad species preferred ants, while the three frog species avoided them (electronic supplementary material, table S4; figure 5), and *L. sphenocephalus* preferred orthopterans, while the other species avoided ingesting them to varying degrees (electronic supplementary material, table S4; figure 5). However, all five species either preferred or took coleopterans in proportion to environmental abundances, preyed upon miscellaneous non-flying predators more than miscellaneous flying predators and preyed upon miscellaneous flying prey less than environmental abundances would predict (figure 5).

## 4. Discussion

We examined isotopes, stomach contents, morphology and habitat selection to determine how five species of frogs and toads coexist. These five anurans partitioned their resources and habitat in complex ways. Although any particular species differed from each of the others on at least one of these measures, no two species differed significantly from one another on all measures. We also found intraspecific variation in niche that may influence interspecific competition.

### 4.1. Niche partitioning

Overall, species varied in trophic level, as indicated by δ^15^N values. In general, larger species had enriched δ^15^N values, indicating that they occupied higher trophic positions [68,69]. *Lithobates catesbeianus,* the species with the longest SVL, had the most enriched δ^15^N values. *Anaxyrus fowleri,* one of the two smallest species, had the most depleted δ^15^N values and thus foraged at the lowest trophic level. *Anaxyrus americanus*, *L. clamitans* and *L. sphenocephalus* occupied similar, intermediate trophic positions but differed in other key ecological factors. Of these three species, *A. americanus* had the smallest SVL and foraged farthest from the ponds; members of this species also took significantly more ants than did either *L. clamitans* or *L. sphenocephalus*. *Lithobates sphenocephalus* differed from *L. clamitans* in that it had a smaller SVL, was found farther from ponds and took more orthopterans*.*

Our focal species also differed significantly in δ^13^C values, which vary depending on the consumption of resources from aquatic (depleted δ^13^C values) versus terrestrial (enriched δ^13^C values) habitats [70]. Both toad species had enriched δ^13^C values, indicating that aquatic prey and habitats were not important foraging areas for them; these results are supported by our habitat selection data, which showed that both toad species were found relatively far from ponds, and by previous studies showing that toads did not visit ponds except for breeding [71,72]. *Lithobates sphenocephalus* was intermediate between the two toads and the other two frog species both in δ^13^C values and in distance from ponds. An earlier study similarly found that *L. sphenocephalus* often ventured into upland habitat and farther from water between breeding periods than *L. catesbeianus* and *L. clamitans* [73]. *Lithobates catesbeianus* frogs had the most depleted δ^13^C values in our study and were found close to ponds and frequently in them. A previous study found that *L. catesbeianus* preyed upon aquatic organisms more frequently than did *L. clamitans* [49], which could explain the more depleted δ^13^C values of *L. catesbeianus*. Another study also found that *L. catesbeianus* individuals were located closer to bodies of water and were more often in water than were *L. clamitans* frogs [55]. By contrast, we found no significant difference in distance to pond between the two species, and we found them in ponds at equal frequencies.

Stomach contents help elucidate niche differences among these ecologically and evolutionarily related species. Ants formed a greater proportion of the diet in the two toad species than in any of the frog species, supporting older studies suggesting that toads in general often prey upon ants more than do most frog species [3,74]. However, according to stomach contents, *A. americanus* is more of a generalist, while *A. fowleri* specializes in ants and coleopterans. *Lithobates sphenocephalus* was the only species to take orthopterans in numbers greater than expected based on orthopteran abundance. Non-flying miscellaneous prey were more frequent than flying miscellaneous prey in the stomachs of all frogs and toads in our study, a bias that may result from a greater difficulty of capturing flying prey.

### 4.2. Intraspecific niche variation

The relationship between isotopic niche and SVL that we observed across species also occurred within species. For δ^15^N values, larger individuals had significantly more enriched δ^15^N values in *A. americanus*, *L. clamitans* and *L. catesbeianus*. Cluster analysis placed most individuals of *L. catesbeianus* in the group with enriched δ^15^N values, but smaller *L. catesbeianus* individuals grouped with larger *A. americanus*, *L. clamitans* and *L. sphenocephalus*. Likewise, smaller individuals of *A. americanus*, *L. clamitans* and *L. sphenocephalus* overlapped in group number with *A. fowleri*, the only species in which most individuals were clustered in the lowest group. This intraspecific niche variation caused by body size can potentially alter competitive interactions and affect niche partitioning [25,40,42,55].

In the case of *L. catesbeianus* at our study site, for example, newly adult frogs will overlap in trophic level with several sympatric species of frogs and toads. However, once *L. catesbeianus* individuals reach approximately 120 mm SVL, they begin to occupy a higher trophic level than the other species and are relatively free from trophic overlap with them. Similarly, in the other study species, competitive interactions will change across the adult phase of their life history, given that early in their lives they will not be in competition with the same species as later in their life. Since only a proportion of the population is therefore competing with heterospecifics, the interaction strength of that competition is probably reduced, which would facilitate niche partitioning and species coexistence.

The relationship between individual size and trophic level in these frogs and toads is not as simple as it might seem. The immediate explanation would be that as individuals become longer, they eat larger prey and those larger prey should be higher on the trophic level. For many vertebrate taxa, there is a large difference in the body sizes of predators and their prey, but these differences in body size are not as large or consistent in invertebrates [75]. Many of the largest arthropods in our study sites were grasshoppers, which are primary consumers (C. S. Cloyed, personal observations), and grasshoppers were ingested most frequently by *L. sphenocephalus*, the smallest true frog. Thus, we speculate that larger anurans in our study may not merely be selecting larger prey but selecting prey that are themselves predators, such as predacious beetles and adult dragonflies.

Intraspecific niche variation associated with individual size may result from both ontogenetic change and individual variation. While frogs are indeterminate growers and longer individuals are often older, tadpole development and size at metamorphosis also influence adult size [76,77]. Faster-developing tadpoles may be shorter at metamorphosis and subsequently throughout their mature stages of life [78]. As a result, the intraspecific variation in size in our study species may be caused by both the effects of ontogeny in indeterminate growers and inter-individual morphological differences associated with tadpole development and metamorphosis. This may be especially true of *L. catesbeianus,* a species in which individuals that overwinter as tadpoles have very large body sizes at metamorphosis [78]. However, further studies are needed to understand the link between tadpole development and size at metamorphosis and how size at metamorphosis may generate variation in the diets of adult frogs and toads.

Cluster analysis showed that species differed more in δ^13^C values than they did in δ^15^N values, but for carbon, anuran distance from pond drove the grouping patterns more than did individual size. However, larger individuals of both *A. americanus* and *L. catesbeianus* had enriched δ^13^C values. In the smaller individuals, a small amount of the isotopes in their skin may have remained from the aquatic tadpole phase, as the half-life of carbon isotopes in frog skin is about 90 days [79]. In larger individuals, any isotopic memory from the tadpole stage or the post-metamorphic stage will have been lost [80–82]. Although in *L. sphenocephalus* the relationship between δ^13^C values and SVL was not significant overall, at several regions larger individuals similarly tended to have enriched δ^13^C values, suggesting that, in this species too, frogs moved away from aquatic habitats as they grew. *Lithobates clamitans* individuals, on the other hand, may continue to use more aquatic habitats and prey as they grow, given that this species showed no relationship between SVL and δ^13^C values.

In our system, stomach content and stable isotope analyses provided unique perspectives on anuran diets and together aided in understanding the multifaceted niche partitioning that can occur in natural populations. In systems in which researchers can compare the same prey groups between stomach contents and stable isotope analysis, results from the two kinds of analyses are frequently similar [83,84]. However, such direct comparisons are often not possible, and consequently other researchers have found that using both techniques provides two differing views of diet or habitat use [85,86]. SCA can provide not only short-term diet information [87,88], but also much more detailed taxonomic information about prey [85–87]. Stable isotopes can help determine trophic structures [68] and define sources of carbon when habitats and diets vary in their δ^13^C values [70]. Hence both techniques are often necessary to provide the more nuanced and complete picture of animals' diets that is needed to understand and predict species interactions and the coexistence of ecologically similar species.

Our study highlights not only how these species can partition their resources through a combination of differences in diet and habitat use but also highlights how intraspecific niche variation can aid partitioning. This is most evident with trophic levels and δ^15^N values. In general, larger species and larger individuals within species forage at higher trophic levels than smaller species and smaller individuals within species. This pattern results in partial species overlap in trophic niche space, with smaller individuals of large species overlapping with larger individuals of small species. Where species largely overlap in resource use, this type of intra-population niche variation can facilitate coexistence because it reduces the potential competition among species by decreasing the number of individuals from each species that overlap in resource use. More work on resource partitioning should begin to investigate how intra-population niche variation contributes to species coexistence.

## Supplementary Material

The supplementary material provides additional information regarding number of frogs and toads captured in different regions, a map of the field site, model selection analysis for the GLMMs performed for each isotope type, additional stomach content analysis and cluster analysis on the stable isotopes.

## Data accessibility

Our data have been deposited at Dryad: http://dx.doi.org/10.5061/dryad.jh843 [89].
