# Supplementary material for: Niche partitioning and the role of intraspecific niche variation in structuring a guild of generalist anurans
Source: R Soc Open Sci. 2017 Mar 15;4(3):170060. doi: 10.1098/rsos.170060 (PMC5383860; doi:10.1098/rsos.170060)
Supplement: The supplementary material provides additional information regarding number of frogs and toads captured in different regions, a map of the field site, model selection analysis for the GLMMs performed for each isotope type, additional stomach content analysis and cluster analysis on the stable isotop [file rsos170060supp1.docx]

Supplementary Tables and Figures

| Variable | PCA 1 Values | PCA 2 Values |
| --- | --- | --- |
| Gape Width | -0.487 | 0.000 |
| Mandible Length | -0.246 | -0.308 |
| Femur Length | 0.391 | 0.489 |
| Tibia Length | 0.472 | 0.000 |
| Metatarsal Length | 0.247 | -0.792 |
| Leg Length | 0.515 | -0.167 |
|  |  |  |
| % Variance Explained | 17% | 17% |

Table S1: Principle Component Analysis for size-corrected morphological traits. Each morphological variable is standardized for snout-vent length.

| Species | Region | N | Average SVL (mm) (±SD) | δ^13^C (±SD) | δ^15^N  (±SD) |
| --- | --- | --- | --- | --- | --- |
| *Anaxyrus americanus* |  |  |  |  |  |
|  | North | 18 | 58.7 (9.3) | -22.8 (0.6) | 3.4 (0.6) |
|  | North Central | 10 | 44.4 (9.1) | -23.5 (0.4) | 3.0 (0.4) |
|  | South Central | 15 | 50.5 (7.8) | -23.0 (0.7) | 3.8 (0.7) |
|  | South | 10 | 50.5 (7.8) | -23.4 (0.4) | 2.0 (0.5) |
|  |  |  |  |  |  |
| *Anaxyrus fowleri* |  |  |  |  |  |
|  | North | 10 | 49.4 (8.2) | -23.2 (0.6) | 2.9 (0.5) |
|  | North Central | 8 | 52.0 (7.7) | -23.1 (0.7) | 2.8 (0.4) |
|  | South Central | 8 | 54.4 (11.8) | -23.3 (0.6 | 3.3 (0.3) |
|  | South | 12 | 53.1 (8.0) | -23.3 (0.5) | 1.8 (0.8) |
|  |  |  |  |  |  |
| *Lithobates catesbeianus* |  |  |  |  |  |
|  | North | 10 | 108.4 (36.8) | -24.9 (1.7) | 4.7 (0.8) |
|  | North Central | 14 | 113.3 (24.8) | -25.0 (0.9) | 4.1 (0.6) |
|  | South Central | 31 | 110.9 (26.2) | -25.1 (1.1) | 4.4 (0.7) |
|  | South | 6 | 108.8 (22.3) | -25.9 (1.5) | 4.3 (0.5) |
|  |  |  |  |  |  |
| *Lithobates clamitans* |  |  |  |  |  |
|  | North | 23 | 77.6 (8.2) | -24.1 (0.4) | 3.0 (0.4) |
|  | North Central | 19 | 79.5 (6.3) | -24.2 (0.4) | 3.2 (0.5) |
|  | South Central | 25 | 76.7 (7.0) | -24.3 (0.4) | 3.3 (0.4) |
|  | South | 8 | 80.2 (6.5) | -24.2 (0.4) | 3.3 (0.6) |
|  |  |  |  |  |  |
| *Lithobates sphenocephalus* |  |  |  |  |  |
|  | North | 7 | 65.8 (10.8) | -23.5 (0.5) | 3.3 (0.4) |
|  | North Central | 11 | 63.7 (8.9) | -23.8 (0.7) | 3.2 (0.5) |
|  | South Central | 16 | 64.2 (8.3) | -23.9 (0.5) | 3.2 (0.5) |
|  | South | 5 | 63.7 (12.0) | -24.0 (0.5) | 3.5 (0.6) |

Table S2: Average snout-vent lengths (SVL), δ^13^C values, and δ^15^N values for each species in each region.

|  | | Model | AICc |
| --- | --- | --- | --- |
| δ^15^N |  | |  |
|  | | δ^15^N ~ Species + SVL | 519.20 |
|  | | δ^15^N ~ Species + SVL + Season | 520.74 |
|  | | δ^15^N ~ Species + SVL + Sex + Season | 522.89 |
|  | | δ^15^N ~ Species + SVL + Sex + Year + Season | 527.93 |
|  | | δ^15^N ~ Species + SVL + Sex + Year + PCA 1 + Season | 532.65 |
|  | | δ^15^N ~ Species + SVL + Sex + Year + PCA 1 + PCA 2 + Season | 537.79 |
|  | | δ^15^N ~ Species | 538.67 |
|  | | δ^15^N ~ SVL | 547.47 |
|  | | δ^15^N ~ Species + SVL + Sex + Year + PCA 1 + PCA 2 + Season + Species*SVL | 572.49 |
|  | | δ^15^N ~ Species + SVL + Sex + Year + PCA 1 + PCA 2 + Season + Species*SVL + Species*Season | 578.06 |
|  | |  |  |
| δ^13^C | |  |  |
|  | | δ^13^C ~ Species + SVL | 580.60 |
|  | | δ^13^C ~ Species + SVL + Sex | 585.57 |
|  | | δ^13^C ~ Species + SVL + Sex + Year | 589.25 |
|  | | δ^13^C ~ Species + SVL + Sex + Year + PCA 1 | 595.25 |
|  | | δ^13^C ~ Species + SVL + Sex + Year + PCA 1 + PCA 2 | 600.48 |
|  | | δ^13^C ~ Species + SVL + Sex + Year + PCA 1 + PCA 2 + Season | 603.49 |
|  | | δ^13^C ~ Species | 609.55 |
|  | | δ^13^C ~ Species + SVL + Sex + Year + PCA 1 + PCA 2 + Season + Species*SVL | 609.63 |
|  | | δ^13^C ~ Species + SVL + Sex + Year + PCA 1 + PCA 2 + Season + Species*SVL + Species*Season | 642.27 |
|  | | δ^13^C ~ SVL | 738.63 |

Table S3: General linear mixed effects model selection for δ^15^N and δ^13^C. For each isotope, the model with the lowest AIC value was used as the final model.

| Species Interaction | Orthoptera | Coleoptera | Ants | Misc. Flying | Misc. Non-Flying |
| --- | --- | --- | --- | --- | --- |
| Anam-Anfo | \| t=-0.350 \| \| --- \| \| *p*=0.363 \| | \| **t=2.307** \| \| --- \| \| ***p*=0.007** \| | \| t=-0.3443 \| \| --- \| \| *p*=0.326 \| | \| **t=-3.762** \| \| --- \| \| ***p*<0.001** \| | \| **t=-5.027** \| \| --- \| \| ***p*=<0.001** \| \|  \| |
| Anam-Lica | \| t=0.0021 \| \| --- \| \| *p*=0.494 \| | \| t=0.001 \| \| --- \| \| *p*=0.495 \| | \| **t=-14.031** \| \| --- \| \| ***p*<0.001** \| | \| **t=2.9** \| \| --- \| \| ***p*=0.001** \| | \| **t=1.96** \| \| --- \| \| ***p*=0.019** \| \|  \| |
| Anam-Licl | \| **t=3.028** \| \| --- \| \| ***p*=0.001** \| | \| **t=2.371** \| \| --- \| \| ***p*=0.006** \| | \| **t=-10.786** \| \| --- \| \| ***p*<0.001** \| | \| **t=3.226** \| \| --- \| \| ***P*<0.001** \| | \| t=1.336 \| \| --- \| \| *p*=0.077 \| \|  \| |
| Anam-Lisp | \| **t=3.552** \| \| --- \| \| ***P*<0.001** \| | \| t=-0.506 \| \| --- \| \| *p*=0.315 \| | \| **t=-5.968** \| \| --- \| \| ***p*<0.001** \| | \| t=-0.42 \| \| --- \| \| *p*=0.346 \| | \| **t=1.511** \| \| --- \| \| ***p*=0.056** \| \|  \| |
| Anfo-Lica | \| t=0.326 \| \| --- \| \| *p*=0.375 \| | \| t=-1.079 \| \| --- \| \| *p*=0.143 \| | \| **t=-13.152** \| \| --- \| \| ***p*<0.001** \| | \| **t=2.742** \| \| --- \| \| ***p*=0.002** \| | \| **t=4.451** \| \| --- \| \| ***p*<0.001** \| \|  \| |
| Anfo-Licl | \| **t=3.405** \| \| --- \| \| ***p*<0.001** \| | \| **t=-2.031** \| \| --- \| \| ***p*=0.028** \| | \| **t=-9.923** \| \| --- \| \| ***p*<0.001** \| | \| **t=4.619** \| \| --- \| \| ***p*<0.001** \| | \| **t=4.117** \| \| --- \| \| ***p*<0.001** \| \|  \| |
| Anfo-Lisp | \| **t=3.707** \| \| --- \| \| ***p*<0.001** \| | \| **t=-3.27** \| \| --- \| \| ***p*=0.002** \| | \| **t=-5.522** \| \| --- \| \| ***p*<0.001** \| | \| t=1.244 \| \| --- \| \| *p*=0.098 \| | \| **t=2.739** \| \| --- \| \| ***p*=0.002** \| \|  \| |
| Lica-Licl | \| **t=3.088** \| \| --- \| \| ***p*<0.001** \| | \| t=-0167 \| \| --- \| \| *p*=0.458 \| | \| **t=2.997** \| \| --- \| \| ***p*=0.001** \| | \| t=-0.272 \| \| --- \| \| *p*=0.417 \| | \| **t=-5.803** \| \| --- \| \| ***p*<0.001** \| \|  \| |
| Lica-Lisp | \| **t=3.577** \| \| --- \| \| ***p*<0.001** \| | \| **t=-2.1** \| \| --- \| \| ***p*=0.025** \| | \| t=1.159 \| \| --- \| \| *p*=0.116 \| | \| **t=-4.597** \| \| --- \| \| ***p*<0.001** \| | \| t=-1.642 \| \| --- \| \| *p*=0.06 \| \|  \| |
| Licl-Lisp | \| **t=2.307** \| \| --- \| \| ***p*=0.007** \| | \| **t=-1.995** \| \| --- \| \| ***p*=0.038** \| | \| t=-0.392 \| \| --- \| \| *p*=0.368 \| | \| **t=-4.271** \| \| --- \| \| ***p*<0.001** \| | \| t=0.922 \| \| --- \| \| *p*=0.159 \| \|  \| |

Table S4: Results from between-species permutation tests of Chesson’s alpha selectivity index. Probability values have been Bonferroni adjusted. Negative t values indicate that the latter species in the listed pair takes fewer of a given prey group than does the first species. Anam= *Anaxyrus americanus,* Anfo= *A. fowleri*, Lica= *Lithobates catesbeianus*, Licl= *L. clamitans*, Lisp= *L. sphenocephalus*.


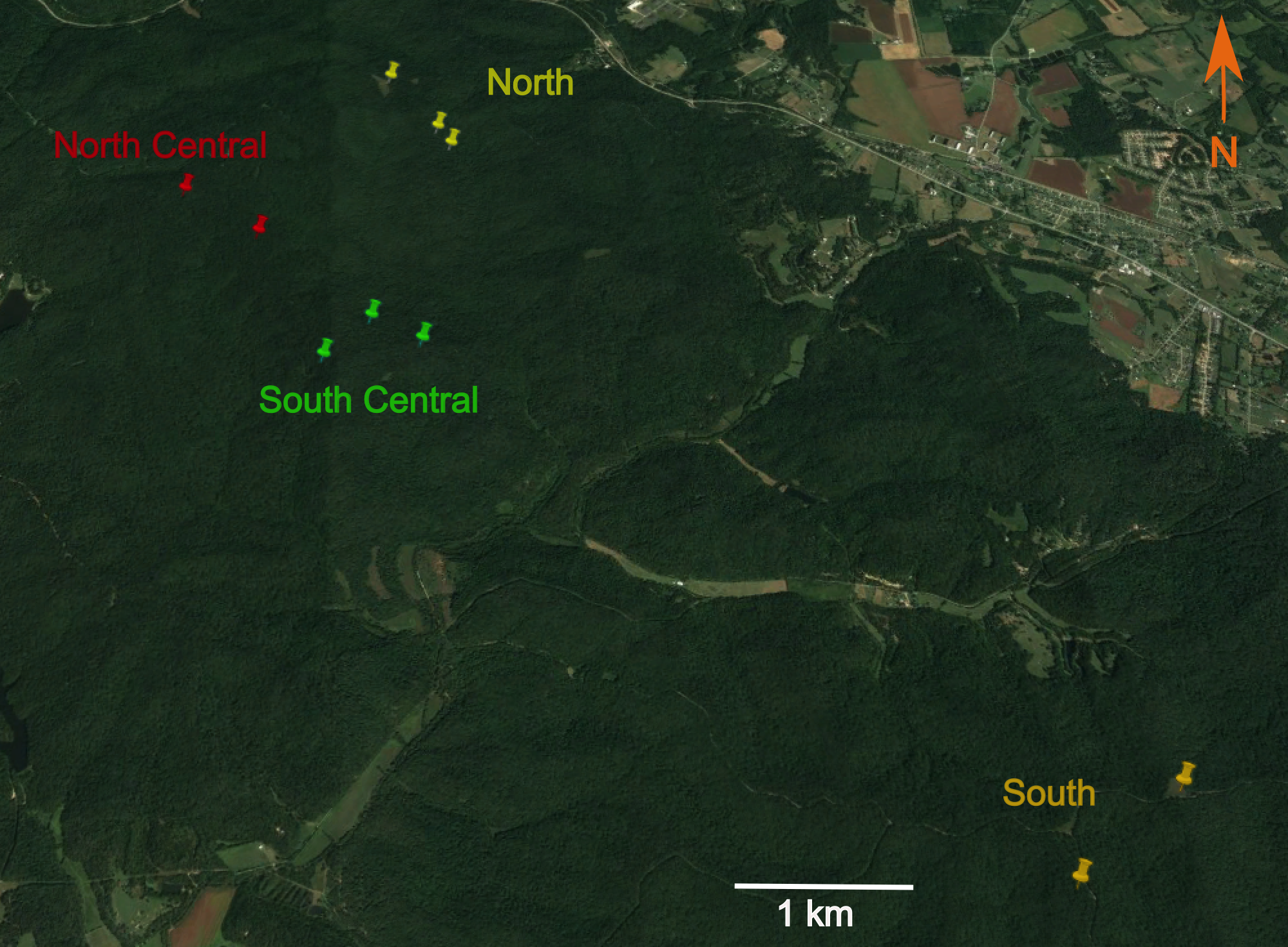


Figure S1: Map of Bernheim Arboretum and Research Forest, with pins representing sampled ponds in each region: north (yellow), north central (red), south central (green), and south (orange). Made with Google® maps.

a)

b)

Figure S2: a) Snout-vent length (mm) of frogs and toads belonging to δ^15^N groups determined by clustering analysis. Individuals in higher-numbered groups have longer snout-vent lengths. b) The distances to pond edge (m) from the locations at which frogs and toads of different δ^13^C groups were found.
